# Absence of Azole Antifungal Resistance in *Aspergillus fumigatus* Isolated from Root Vegetables Harvested from UK Arable and Horticultural Soils

**DOI:** 10.3390/jof6040208

**Published:** 2020-10-06

**Authors:** Mireille H. van der Torre, Cheryl Whitby, Christopher P. Eades, Caroline B. Moore, Lilyann Novak-Frazer, Malcolm D. Richardson, Riina Rautemaa-Richardson

**Affiliations:** 1 Mycology Reference Centre Manchester, ECMM Centre of Excellence for Medical Mycology, Manchester University NHS Foundation Trust, Wythenshawe Hospital, Manchester M23 9LT, UK; mireille.vandertorre-2@manchester.ac.uk (M.H.v.d.T.); cheryljwhitby@icloud.com (C.W.); Caroline.Moore@manchester.ac.uk (C.B.M.); Lily.Novak-Frazer@manchester.ac.uk (L.N.-F.); malcolm.richardson@manchester.ac.uk (M.D.R.); 2 Division of Infection, Inflammation and Respiratory Medicine, Faculty of Biology, Medicine and Health, University of Manchester, Manchester M13 9NT, UK; christopher.eades@manchester.ac.uk; 3 Department of Infectious Diseases, Manchester University NHS Foundation Trust, Wythenshawe Hospital, Manchester M23 9LT, UK

**Keywords:** *Aspergillus fumigatus*, azole resistance, environmental sampling, soil

## Abstract

The emergence of azole-resistant *Aspergillus fumigatus* (AR*Af*) complicates the treatment of aspergillosis and can nearly double the mortality from invasive aspergillosis (IA). AR*Af* has been isolated from many different environmental sites and indoor environments and thus presents a significant risk for susceptible patients. Local surveillance of environmental AR*Af* can guide antifungal prescribing and improve patient outcomes. In this study, seventy-four soils samples collected from the surface of a variety of root vegetables from farm shops and private gardens covering a wide geographical area of the UK, were cultured to assess the presence of *A. fumigatus,* and the prevalence and nature of any resistance mechanisms. A high-throughput in-house antifungal susceptibility screening method was developed and validated using the EUCAST MIC reference method, E.DEF 9.3.1. A total of 146 isolates were recovered and analysed. Even though the study premise was that soil-covered root vegetables and other fresh produce could represent a conduit for AR*Af* exposure in vulnerable patients, no AR*Af* were found in the soil samples despite 55% of samples harbouring *A. fumigatus.* The sample type and screening method used could be suitable for more extensive monitoring of the soil to detect trends in the prevalence of AR*Af.*

## 1. Introduction

Saprophytic moulds of the genus *Aspergillus*, mainly found in various horizons of soil (humus), leaf litter and decomposing plant matter [1], cause a variety of fungal diseases in crops, animals and humans [2,3,4,5]. The production of airborne spores and their ability to adapt to a wide range of environmental conditions play an important role in pathogenesis [6]. This adaptation extends to human pathogenesis, particularly of immunocompromised patients or those with underlying lung conditions. Aspergillosis, commonly caused by *A. fumigatus*, affects millions of people worldwide as allergic, invasive or chronic disease, which can be fatal especially when treatment is not effective [7]. Triazole antifungals, including itraconazole, voriconazole, and posaconazole, are the first line choice of drugs in the treatment and prophylaxis of aspergillosis [8]. Resistance to one or more of the azole antifungals is an increasing clinical problem, and is associated with prolonged and low dose use of these drugs (within patient resistance development) and with extensive agricultural use of azole fungicides (environmental resistance development) [9,10,11]. Invasive infections caused by azole-resistant *A. fumigatus* (AR*Af*) have a significantly higher mortality rate compared to those with azole-susceptible *A. fumigatus* [12]. Azole antifungal resistance is often associated with an alteration of the drug target lanosterol 14-α-sterol demethylase, a key enzyme in the ergosterol biosynthesis pathway, encoded by the gene, *cyp51A*. Specific point mutations in *cyp51A,* which may occur in association with tandem repeats in the promoter region of the gene, have been suggested to be the most common azole resistance mechanisms [13]. 

A recent German study reported a lack of evidence to support environmental resistance development due to extensive fungicide use as no increased incidence of AR*Af* was found in fields that were actively treated with azole fungicides [14]. Regardless of whether the rise in medical AR*Af* originates from environmental overuse, two mechanisms of resistance are predominantly linked to environmental AR*Af*: a 34-bp-long tandem repeat in the promoter region combined with a leucine-to-histidine change at codon 98 (TR34/L98H) or TR46/Y121F/T289A [10,15,16]. Worryingly, a high proportion of azole-resistant isolates possessing the TR34/L98H mutation have been recovered from patients with no history of prior azole treatment, suggesting that resistant strains have been acquired from the environment [12]. To test this hypothesis, a hospital indoor environmental isolate collection, assembled between 1995 and 2007, was analysed and additional soil samples were collected in direct proximity to the Dutch University Medical Centre [15]. In total, *A. fumigatus* was present in 248 indoor environmental samples, including five azole-resistant isolates from patient rooms and hospital water. Of 49 *A. fumigatus-*positive outdoor samples, six were found to be azole-resistant. Interestingly, all AR*Af* were obtained from flower beds but none were found in natural soil.

Numerous environmental surveillance studies have confirmed the existence of environmental AR*Af* worldwide in the last ten years [17,18,19,20,21,22,23,24,25,26]. AR*Af* has been isolated from many different environmental sites including from the air, compost, leaves, seeds, plants, flower bulbs, onions and flower beds, and regularly found near or in hospitals, thus presenting a significant risk for susceptible patients [27,28]. Additionally, some of these studies show a worrying trend of AR*Af* collected from the patients’ own homes, suggesting a wider degree of environmental dissemination [29,30]. Notably, a fatal case of treatment-resistant invasive aspergillosis (due to AR*Af* harbouring the TR46/Y121F/T289A mutations) has been reported in an immunosuppressed patient [31]. In this case, it was established that the clinical and environmental isolates, obtained from the patient’s home, were genetically indistinguishable. 

The emergence of AR*Af* presents an increasing public health problem. To further elucidate its magnitude the European Centre for Disease Prevention and Control (ECDC) advocates active surveillance [32]. To date, only a small number of studies have been performed in the UK with varying recovery rates among different sampling regions. The 2009-2011 environmental prevalence study in Greater Manchester (in the North West of England) detected AR*Af* in 4/231 (1.7%) of rural *A. fumigatus* isolates but none in urban environments [33]. On the contrary, the prevalence of AR*Af* in South Wales was found to be 5.2% in agricultural areas and 8.4% in urban areas [34]. Conversely, of the 74% *A. fumigatus*-positive soil samples from Southern England, the prevalence of resistance was highest in urban samples [35]. The findings of these data suggest that azole resistance can vary significantly between sampling sites across relatively small geographical areas. Therefore, the aim of this study was to classify *Aspergillus fumigatus* isolates from arable sites across the UK in order to assess the prevalence and nature of any resistance mechanisms. By sampling the soil adhering to root vegetables (soil transporters) acquired from UK farms and private gardens, we also sought to assess whether fresh produce could represent a conduit for AR*Af* exposure in the living environment of vulnerable outpatients.

## 2. Materials and Methods

### 2.1. Environmental Sampling and Isolation of Aspergillus Species

Soil samples adhering to a variety of root vegetables (including potatoes, carrots, parsnips, beetroot and Jerusalem artichokes) were collected from 18 farms or private gardens across 11 counties in the UK during 2018. The soil was collected by swabbing the outside of the vegetable with sterile distilled water. The swab containing the soil was then suspended in two ml of sterile, distilled water and vortexed thoroughly. Subsequently, one mL of this suspension was plated onto Sabouraud (SAB) dextrose agar with chloramphenicol, and incubated at 45 °C to maximize the selective yield for *Aspergillus* species. The identification of filamentous fungi was based on examination of their macroscopic and microscopic morphology and by DNA sequencing when required (see Section 2.3. Molecular Identification of Azole-Resistant Fungal Species).

### 2.2. Susceptibility Testing

All environmental isolates grown on the SAB plates were primary screened for azole resistance using a 24-well plate in-house screening method. A standardised inoculum (two to five ×10^5^ conidia/mL) was used on the screening plates containing RPMI agar medium supplemented with azole antifungals at a concentration in keeping with the current European Committee on Anti-Microbial Susceptibility Testing (EUCAST) therapeutic breakpoints for minimum inhibitory concentration (MIC): Itraconazole (ITC) (2 mg/L); voriconazole (VRC) (1 mg/L), posaconazole (POS) (0.25 mg/L); or no antifungal drug (positive control). The screening plates were incubated at 37 °C, and end-points were recorded at 48 h for itraconazole and voriconazole, and at 72 h for posaconazole to reduce false negative results. Any growth, including poor growth, was recorded as a positive screening result; no growth was recorded as a negative result. Isolates that were potentially azole resistant were tested further according to the EUCAST micro-broth dilution reference method [36]. The isolates were considered resistant when at least one MIC exceeded the EUCAST breakpoints. 

### 2.3. Molecular Identification of Azole-Resistant Fungal Species

To extract genomic DNA from the azole-resistant (as defined above) culture isolates, a small amount of hyphae (approximately 2 ± 0.5 mm^2^, from the outermost part of the culture) was harvested using a disposable 10 µL loop and transferred into a 1.5 mL screw-cap microcentrifuge tube containing acid-washed glass beads (G8772, Sigma-Aldrich, now part of Merck, St. Louis, MO, USA) and 200 µL PrepMan™ Ultra Sample Preparation Reagent (ThermoFisher Scientific Inc., Waltham, MA, USA). The tubes were vortexed thoroughly for 10 secs and incubated for 10 min at 100 °C before bead-beating using a MagNa Lyser™ (Roche Diagnostics GmBH, Basel, Switzerland) for 70 s at 7000 rpm. The samples were centrifuged for 15 s at 13,000 rpm, following which another cycle of heating and bead-beating was performed. Finally, the tubes were centrifuged for 1 min at 13,000 rpm, and the supernatant containing genomic DNA was used in a polymerase chain reaction (PCR) to amplify genes encoding the internal transcribed spacer regions (ITS), β-tubulin (β*-tub*) and calmodulin (*CaM*). The ITS primers span the variable regions located between the conserved genes encoding the 18S and 26-28S rRNA subunits, a common region for sequence-based fungal identification [37]. Particularly for *Aspergillus* spp., other genes are required for further DNA-based discrimination [38].

Amplification reactions were each performed in a final volume of 25 μL containing 10 pmol each of forward and reverse primers, 1× HotStarTaq^®^ *Plus* master mix (Qiagen N.V., Hilden, Germany) and 1 μL of genomic DNA. The primer sequences and cycling conditions for ITS5/ITS4 [39], Bt2a/Bt2b [40] and Cal5F/Cal6R [41] were used as described previously. The PCR products were checked using agarose gel electrophoresis, then purified using the QIAquick^®^ PCR purification kit (Qiagen N.V., Hilden, Germany) in accordance with the manufacturer’s instructions. The purified PCR products were sent to Eurofins Genomics GmbH for sequencing. Sequence reads were aligned, and the alignment data used to search public DNA sequence databases (National Centre for Biotechnology Information (NCBI); The Westerdijk Institute; and the International Society for Human and Animal Mycology (ISHAM)) to determine fungal identity. Consensus among the ITS, β*-tub* and *CaM* matches as well as among the three databases used confirmed the identity of each isolate.

## 3. Results

### 3.1. Environmental Sampling

**Figure 1:**
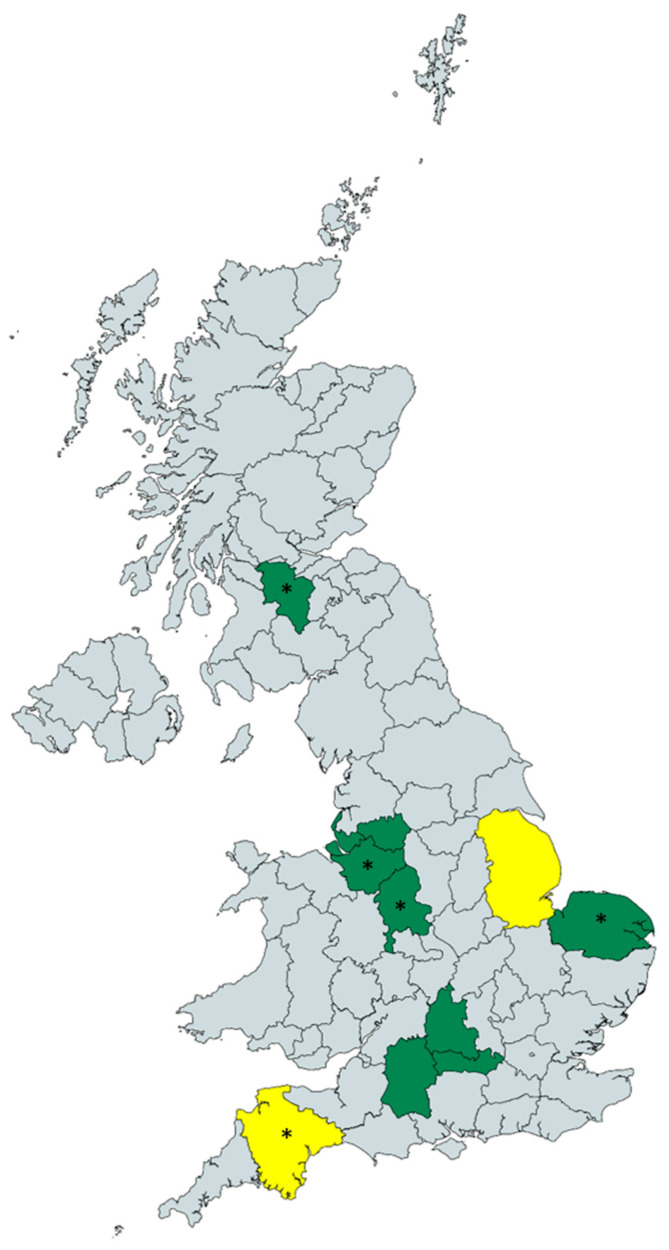
UK map showing the areas included in the study. Unwashed root vegetables (*n* = 74) were collected from 18 different farm shops and *A. fumigatus* were recovered from the green highlighted counties, whereas no *A. fumigatus* growth was detected from samples collected in the yellow highlighted counties. Azole-resistant non-*fumigatus* spp. were recovered from the counties with an asterisk. The map was created using mapchart.net.

Seventy-four samples of soil adhering to root vegetables (soil transporters) sold at 18 different farm shops around the UK, covering a wide geographical area (Figure 1). Samples were collected from Berkshire (*n* = 9), Cheshire (*n* = 28), Devon (*n* = 3), Greater Manchester (*n* = 3), Lincolnshire (*n* = 3), Merseyside (*n* = 3), Norfolk (*n* = 6), Oxfordshire (*n* = 6) South Lanarkshire (*n* = 1), Staffordshire (*n* = 9) and Wiltshire (*n* = 3) and included soil from potatoes, carrots, parsnips, beetroot and Jerusalem artichokes. Of these, 41 (55%) grew *A. fumigatus* with a total of 146 *A. fumigatus* isolates recovered overall (Table 1). Among these samples, no azole-resistant strains were detected.

### 3.2. Antifungal Susceptibility Testing of Aspergillus Species and Their Identification

A high-throughput, in-house antifungal susceptibility screening method was developed and validated using the EUCAST MIC reference method (E.DEF 9.3.1) resulting in an overall sensitivity and specificity using all three drugs in combination (ITC, VRC and POS) of 94% and 100%, respectively [36]. 

No AR*Af* were found during the initial screens. However, six non-*fumigatus* aspergilli with resistance to at least one of the antifungal azole drugs were detected using the 24-well plate screening method. These isolates were tested further using the EUCAST broth-dilution reference method, and identified by multi-locus comparison of their ITS, β *-tub* and *CaM* sequences as members of *Aspergillus* Section *Fumigati* (Table 2) [38]. Three isolates were identified as *Neosartorya fischeri*, the teleomorph of *Aspergillus fischeri*, one as *Neosartorya spinosa* (basionym of *Aspergillus fischeri* var. *spinosus*, now known as *A. spinosus*) and two as *Aspergillus oerlinghausenensis*, a species closely related to *A. fumigatus* [42,43]. All six isolates were found to be pan-azole resistant (Table 2) [44].

## 4. Discussion

The emerging azole resistance of *A. fumigatus* is a global challenge to clinical care. Environmental monitoring for AR*Af* is important to fully understanding its prevalence and impact at the local level. Our study did not find AR*Af* in samples collected from the soil adhering to root vegetables despite the fact that 55% were positive for *A. fumigatus* complex growth, with a total of 146 isolates recovered. These data concur with two soil surveys performed in 2010 and 2013 in Denmark, where an absence of environmental AR*Af* was reported [45]. Our data are also in agreement with a recent study in Germany that reported an overall low incidence of AR*Af* in agricultural samples, despite sampling in fields before and after azole fungicide treatment [14]. Two local studies in the UK reported the occurrence of environmental AR*Af*, but with a clear difference between urban and rural locations. The prevalence of AR*Af* in urban areas of South Wales and Southern England was notably higher than in agricultural areas [34,35]. Conversely, data from a similar study conducted across Greater Manchester in 2014 found a higher prevalence of AR*Af* in agricultural areas with previous history of azole use, albeit still as low as 1.7%, compared to none in the urban areas [33]. That azole resistance seems to be correlated with urban areas in the UK is a significant worry for susceptible patients. Although since our study found no *A. fumigatus* growth nor AR*Af* in widely dispersed geographical locations of the UK suggests that root vegetables are not likely to be the cause of transmission of *A. fumigatus* triazole resistance.

A noteworthy finding of this study is the isolation of six mould isolates with azole resistance that are very closely related to *A. fumigatus.* The heat-resistant species *N. fischeri* and *N. spinosa* are associated with spoilage of food processed by heating and previously detected in soil studies including Greater Manchester [33,46,47,48]. Interestingly, we also identified two resistant *A. oerlinghausenensis* isolates [43]. This species has only recently been described following its isolation in German soil in 2015 [26] and from French sawmills in 2017 [49]. No reports on azole-susceptible *A. oerlinghausenensis* have been published yet, suggesting that this species might have intrinsic azole-resistant properties, as seen in several sibling species of *A. fumigatus* such as *A. lentulus* and *A. udagawae* [50]. Further molecular analysis is crucial to identify possible other molecular mechanisms leading to its azole resistance.

In this study, all isolates were tested initially for antifungal susceptibility through our in-house screening protocol based on the use of azole-containing agar plates, and confirmed by the EUCAST liquid-based, reference methodology [36]. Our protocol was optimised using 60 isolates from our archive of *A. fumigatus* complex-positive clinical specimens, and tested at a variety of incubation times, drug concentrations and temperatures to increase screening sensitivity. In addition, our protocol uses half-concentrations of azole antifungals (versus a previous validated screening method and the commercially available VIPCheck^TM^ system (Mediaproducts B.V., Groningen, The Netherlands)) to reduce the amount of false negatives and to detect any isolates with reduced azole susceptibility [34,51]. Since our screening method was validated using *A. fumigatus* isolates, the suitability of this protocol for non-*fumigatus* moulds is uncertain. While susceptibility testing of these isolates was also undertaken using the broth-dilution method, the prevalence of azole resistant non-*fumigatus* isolates could have been undervalued using this method given that breakpoints are only validated for species that cause clinically significant invasive fungal disease [36].

A limitation of the study resides in the lack of data concerning fungicide use in some of the areas from which the samples were obtained. Previously published data indicate an association between AR*Af* and the use of agricultural azoles [9,15,33,52], except for the recent German study [14]. It has recently been emphasised that there is evidence fungicide use is not driving resistance [14]. Other observations suggest that the development of azole resistance in *A. fumigatus* and other environmental moulds is heterogeneous, and may depend on the precise agricultural azole(s) used, the duration of exposure and the concentration of azole(s) at the site of sampling [9,10,49]. However, while various fungicides used to control crop disease include products that contain azoles, the use of products that contain strobilurins are more often applied for disease control of vegetable crops [53,54]. Another limitation of this study is the uneven distribution of the sample sites throughout the UK, where Cheshire is overrepresented compared to a lower number of samples from elsewhere. Moreover, some of the samples were retrieved from farm shops, and therefore from a second location from where they were grown. It is subsequently possible that airborne conidia landed on the surface of the vegetables and adhering soil in the farm shop and were isolated during the process.

This study was designed to provide a snapshot of the prevalence of resistant *A. fumigatus*, and it is, therefore, not possible to determine whether there is temporal variation in the prevalence of AR*Af* in the soils sampled—as has been suggested by several European sources. Indeed, AR*Af* was detected in Denmark during the summer months, whereas no AR*Af* was isolated from samples taken during spring and autumn [28,45]. Although the airborne conidia of *A. fumigatus* were not found to be correlated with temperature [55], resistance prevalence was not measured and thus it might be possible that weather conditions play a significant role in AR*Af* prevalence. 

Soil on root vegetables has not been shown, or suggested to be, a route for AR*Af* exposure. As this study was one of the few in the UK, we recommend further and more extensive monitoring of the soil, including areas with and without azole fungicide exposure, to determine trends in the prevalence of AR*Af.* A recent mycology citizen science project encouraged collection of air and environmental samples from participants’ homes and workplaces worldwide [56]. In total, 7991 *A. fumigatus* isolates were recovered from the returned samples; these will ultimately be tested for azole antifungal-resistance, which may illuminate its global distribution. The analysis of such a large number of samples can be achieved using the multidrug resistance screening method described here since it allows a higher number of isolates to be processed for resistance screening in comparison to the more labour-intensive broth-dilution method. The findings should then be implemented in patient care, for example, by performing resistance screening or reconsidering the use of azoles as the first choice of antifungal drug in aspergillosis patients living in areas with an elevated environmental AR*Af* incidence.

## Figures and Tables

**Table 1:** Origin of soil samples on root vegetable transporters and *A. fumigatus* recovery rates.

Sampling Site	Number of Samples Collected	Number of Samples with *A. fumigatus* Growth (%)	Total *A. fumigatus* Isolates Recovered
Berkshire	9	5 (56)	9
Cheshire	28	15 (54)	34
Devon	3	0 (0)	0
Greater Manchester	3	1 (33)	2
Lincolnshire	3	0 (0)	0
Merseyside	3	3 (100)	15
Norfolk	6	3 (50)	6
Oxfordshire	6	3 (50)	5
South Lanarkshire	1	1 (100)	10
Staffordshire	9	8 (89)	60
Wiltshire	3	2 (67)	5
**Total**	74	41 (55)	146

**Table 2:** Characteristics of the azole-resistant fungal isolates identified in this study.

Isolate	Sampling Site	Sample Source	Azole Resistance Primary				MIC (mg/L) ^2^			Species
			Screening Method Results ^1^							
			Control	ITC	VRC	POS	ITC	VRC	POS	
4	Norfolk	Beetroot	+	-	+	-	>8	4	0.5	*Neosartorya fischeri*
13	Staffordshire	Carrot	+	-	+	-	>8	2	0.5	*Neosartorya fischeri*
99	South Lanarkshire	Potato	+	+	+	-	>8	4	0.5	*Aspergillus oerlinghausenensis*
142	Devon	Potato	+	+	+	+	>8	8	2	*Neosartorya spinosa*
146	Cheshire	Potato	+	+	+	-	>8	4	0.5	*Aspergillus oerlinghausenensis*
148	Cheshire	Potato	+	+	+	-	>8	4	0.5	*Neosartorya fischeri*

^1^ ITC, itraconazole; VRC, voriconazole; POS, posaconazole; ^2^ MIC determinations obtained using EUCAST micro-broth dilution reference method.

## References

[1] Klich M.A. (2002). Biogeography of *Aspergillus* species in soil and litter. Mycologia.

[2] Perrone G., Susca A., Cozzi G., Ehrlich K., Varga J., Frisvad J.C., Meijer M., Noonim P., Mahakarnchanakul W., Samson R.A. (2007). Biodiversity of *Aspergillus* species in some important agricultural products. Stud. Mycol..

[3] Seyedmousavi S., Guillot J., Arne P., de Hoog G.S., Mouton J.W., Melchers W.J., Verweij P.E. (2015). *Aspergillus* and aspergilloses in wild and domestic animals: A global health concern with parallels to human disease. Med. Mycol..

[4] Latgé J.P., Chamilos G. (2019). *Aspergillus fumigatus* and Aspergillosis in 2019. Clin. Microbiol. Rev..

[5] Barber A.E., Scheufen S., Walther G., Kurzai O., Schmidt V. (2020). Low rate of azole resistance in cases of avian aspergillosis in Germany. Med. Mycol..

[6] Kwon-Chung K.J., Sugui J.A. (2013). *Aspergillus fumigatus*--what makes the species a ubiquitous human fungal pathogen?. PLoS Path..

[7] Brown G.D., Denning D.W., Gow N.A., Levitz S.M., Netea M.G., White T.C. (2012). Hidden killers: Human fungal infections. Sci. Transl. Med..

[8] Ullmann A.J., Aguado J.M., Arikan-Akdagli S., Denning D.W., Groll A.H., Lagrou K., Lass-Flörl C., Lewis R.E., Munoz P., Verweij P.E. (2018). Diagnosis and management of *Aspergillus* diseases: Executive summary of the 2017 ESCMID-ECMM-ERS guideline. Clin. Microbiol. Infect..

[9] Snelders E., Camps S.M., Karawajczyk A., Schaftenaar G., Kema G.H., van der Lee H.A., Klaassen C.H., Melchers W.J., Verweij P.E. (2012). Triazole fungicides can induce cross-resistance to medical triazoles in *Aspergillus fumigatus*. PLoS ONE.

[10] Ren J., Jin X., Zhang Q., Zheng Y., Lin D., Yu Y. (2017). Fungicides induced triazole-resistance in *Aspergillus fumigatus* associated with mutations of TR46/Y121F/T289A and its appearance in agricultural fields. J. Hazard. Mater..

[11] Bowyer P., Moore C.B., Rautemaa R., Denning D.W., Richardson M.D. (2011). Azole antifungal resistance today: Focus on *Aspergillus*. Current Infect. Dis. Rep..

[12] van der Linden J.W., Snelders E., Kampinga G.A., Rijnders B.J., Mattsson E., Debets-Ossenkopp Y.J., Kuijper E.J., Van Tiel F.H., Melchers W.J., Verweij P.E. (2011). Clinical implications of azole resistance in *Aspergillus fumigatus*, The Netherlands, 2007-2009. Emerg Infect. Dis..

[13] Chowdhary A., Sharma C., Meis J.F. (2017). Azole-Resistant Aspergillosis: Epidemiology, Molecular Mechanisms, and Treatment. J. Infect. Dis..

[14] Barber A.E., Riedel J., Sae-Ong T., Kang K., Brabetz W., Panagiotou G., Deising H.B., Kurzai O. (2020). Azole use in agriculture reduces *Aspergillus fumigatus* abundance but does not alter its population structure. bioRxiv.

[15] Snelders E., Huis In ‘t Veld R.A., Rijs A.J., Kema G.H., Melchers W.J., Verweij P.E. (2009). Possible environmental origin of resistance of *Aspergillus fumigatus* to medical triazoles. Appl. Environment. Microbiol..

[16] Snelders E., van der Lee H.A., Kuijpers J., Rijs A.J., Varga J., Samson R.A., Mellado E., Donders A.R., Melchers W.J., Verweij P.E. (2008). Emergence of azole resistance in *Aspergillus fumigatus* and spread of a single resistance mechanism. PLoS Med..

[17] Vaezi A., Fakhim H., Javidnia J., Khodavaisy S., Abtahian Z., Vojoodi M., Nourbakhsh F., Badali H. (2018). Pesticide behavior in paddy fields and development of azole-resistant *Aspergillus fumigatus*: Should we be concerned?. J. Mycol. Med..

[18] Prigitano A., Esposto M.C., Romanò L., Auxilia F., Tortorano A.M. (2019). Azole-resistant *Aspergillus fumigatus* in the Italian environment. J. Glob. Antimicrob. Re..

[19] Hagiwara D. (2018). Current Status of Azole-resistant *Aspergillus fumigatus* Isolates in East Asia. Med. Mycol. J..

[20] Lockhart S.R., Frade J.P., Etienne K.A., Pfaller M.A., Diekema D.J., Balajee S.A. (2011). Azole resistance in *Aspergillus fumigatus* isolates from the ARTEMIS global surveillance study is primarily due to the TR/L98H mutation in the *cyp51A* gene. Antimicrob. Agents Ch..

[21] Chowdhary A., Sharma C., Kathuria S., Hagen F., Meis J.F. (2014). Azole-resistant *Aspergillus fumigatus* with the environmental TR46/Y121F/T289A mutation in India. J. Antimicrob. Ch..

[22] Le Pape P., Lavergne R.A., Morio F., Alvarez-Moreno C. (2016). Multiple Fungicide-Driven Alterations in Azole-Resistant *Aspergillus fumigatus*, Colombia, 2015. Emerg. Infect. Dis..

[23] Chowdhary A., Sharma C., van den Boom M., Yntema J.B., Hagen F., Verweij P.E., Meis J.F. (2014). Multi-azole-resistant *Aspergillus fumigatus* in the environment in Tanzania. J. Antimicrob. Ch..

[24] Chen Y., Dong F., Zhao J., Fan H., Qin C., Li R., Verweij P.E., Zheng Y., Han L. (2020). High Azole Resistance in *Aspergillus fumigatus* Isolates from Strawberry Fields, China, 2018. J. Antimicrob. Ch..

[25] Hurst S.F., Berkow E.L., Stevenson K.L., Litvintseva A.P., Lockhart S.R. (2017). Isolation of azole-resistant *Aspergillus fumigatus* from the environment in the south-eastern USA. J. Antimicrob. Ch..

[26] Bader O., Tünnermann J., Dudakova A., Tangwattanachuleeporn M., Weig M., Groß U. (2015). Environmental isolates of azole-resistant *Aspergillus fumigatus* in Germany. Antimicrob. Agents Ch..

[27] Godeau C., Reboux G., Scherer E., Laboissiere A., Lechenault-Bergerot C., Millon L., Rocchi S. (2020). Azole-resistant *Aspergillus fumigatus* in the hospital: Surveillance from flower beds to corridors. Am. J. Infect. Control.

[28] Mortensen K.L., Mellado E., Lass-Flörl C., Rodriguez-Tudela J.L., Johansen H.K., Arendrup M.C. (2010). Environmental study of azole-resistant *Aspergillus fumigatus* and other aspergilli in Austria, Denmark, and Spain. Antimicrob. Agents Ch..

[29] Dauchy C., Bautin N., Nseir S., Reboux G., Wintjens R., Le Rouzic O., Sendid B., Viscogliosi E., Le Pape P., Arendrup M.C. (2018). Emergence of *Aspergillus fumigatus* azole resistance in azole-naïve patients with chronic obstructive pulmonary disease and their homes. Indoor Air.

[30] van der Linden J.W., Camps S.M., Kampinga G.A., Arends J.P., Debets-Ossenkopp Y.J., Haas P.J., Rijnders B.J., Kuijper E.J., van Tiel F.H., Varga J. (2013). Aspergillosis due to voriconazole highly resistant *Aspergillus fumigatus* and recovery of genetically related resistant isolates from domiciles. Clin. Infect. Dis..

[31] Lavergne R.A., Chouaki T., Hagen F., Toublanc B., Dupont H., Jounieaux V., Meis J.F., Morio F., Le Pape P. (2017). Home Environment as a Source of Life-Threatening Azole-Resistant *Aspergillus fumigatus* in Immunocompromised Patients. Clin. Infect. Dis..

[32] Bowyer P., Kleinkauf N., Verweij P.E., Arendrup M.C., Donnelly P.J., Cuenca-Estrella M., Fraaije B., Melchers W.J.G., Adriaenssens N., Kema G.H.J. (2013). Risk assessment on the impact of environmental usage of triazoles on the development and spread of resistance to medical triazoles in *Aspergillus* species. Stockholm: ECDC.

[33] Bromley M.J., van Muijlwijk G., Fraczek M.G., Robson G., Verweij P.E., Denning D.W., Bowyer P. (2014). Occurrence of azole-resistant species of *Aspergillus* in the UK environment. J. Global Antimicrob. Res..

[34] Tsitsopoulou A., Posso R., Vale L., Bebb S., Johnson E., White P.L. (2018). Determination of the Prevalence of Triazole Resistance in Environmental *Aspergillus fumigatus* Strains Isolated in South Wales, UK. Front. Microbiol..

[35] Sewell T.R., Zhang Y., Brackin A.P., Shelton J.M.G., Rhodes J., Fisher M.C. (2019). Elevated Prevalence of Azole-Resistant *Aspergillus fumigatus* in Urban versus Rural Environments in the United Kingdom. Antimicrob. Agents Chem..

[36] Rodriquez-Tudela J.L., Donnelly J.P., Arendrup M.C., Arikan S., Barchiesi F., Bille J., Chryssanthou E., Cuenca-Estrella M., Dannaoui E., Denning D. (2008). EUCAST Technical Note on the method for the determination of broth dilution minimum inhibitory concentrations of antifungal agents for conidia-forming moulds. Clin. Microbiol. Infect..

[37] Schoch C.L., Seifert K.A., Huhndorf S., Robert V., Spouge J.L., Levesque C.A., Chen W. (2012). Nuclear ribosomal internal transcribed spacer (ITS) region as a universal DNA barcode marker for Fungi. Proceed. Nat. Acad. Sci. USA.

[38] Peterson S.W. (2008). Phylogenetic analysis of *Aspergillus* species using DNA sequences from four loci. Mycologia.

[39] White T.J., Bruns T., Lee S., Taylor J., Innis M.A., Gelfand D.H. (1990). 38 - Amplification and Direct Sequencing of Fungal Ribosomal RNA Genes for Phylogenetics. PCR Protocols.

[40] Glass N.L., Donaldson G.C. (1995). Development of primer sets designed for use with the PCR to amplify conserved genes from filamentous ascomycetes. Appl. Environment. Microbiol..

[41] Hong S.B., Go S.J., Shin H.D., Frisvad J.C., Samson R.A. (2005). Polyphasic taxonomy of *Aspergillus fumigatus* and related species. Mycologia.

[42] Samson R.A., Hong S., Peterson S.W., Frisvad J.C., Varga J. (2007). Polyphasic taxonomy of *Aspergillus* section *Fumigati* and its teleomorph *Neosartorya*. Stud. Mycol..

[43] Houbraken J., Weig M., Gross U., Meijer M., Bader O. (2016). *Aspergillus oerlinghausenensis*, a new mould species closely related to *A. fumigatus*. FEMS Microbiol. Lett..

[44] Chen L., Krekels E.H.J., Verweij P.E., Buil J.B., Knibbe C.A.J., Brüggemann R.J.M. (2020). Pharmacokinetics and Pharmacodynamics of Posaconazole. Drugs.

[45] Astvad K.M., Jensen R.H., Hassan T.M., Mathiasen E.G., Thomsen G.M., Pedersen U.G., Christensen M., Hilberg O., Arendrup M.C. (2014). First detection of TR46/Y121F/T289A and TR34/L98H alterations in *Aspergillus fumigatus* isolates from azole-naive patients in Denmark despite negative findings in the environment. Antimicrob. Agents Chem..

[46] Hong S.B., Kim D.H., Park I.C., Samson R.A., Shin H.D. (2010). Isolation and identification of *Aspergillus* section *fumigati* strains from arable soil in Korea. Mycobiology.

[47] Ugwuanyi J.O., Obeta J.A. (1991). Incidence of heat-resistant fungi in Nsukka, southern Nigeria. Int. J. Food Microbiol..

[48] Santos J., Samapundo S., Biyikli A., Van Impe J., Akkermans S., Höfte M., Abatih E.N., Sant’Ana A.S., Devlieghere F. (2018). Occurrence, distribution and contamination levels of heat-resistant moulds throughout the processing of pasteurized high-acid fruit products. Int. J. Food Microbiol..

[49] Jeanvoine A., Rocchi S., Reboux G., Crini N., Crini G., Millon L. (2017). Azole-resistant *Aspergillus fumigatus* in sawmills of Eastern France. J. Appl. Microbiol..

[50] Van Der Linden J.W., Warris A., Verweij P.E. (2011). *Aspergillus* species intrinsically resistant to antifungal agents. Med. Mycol..

[51] Arendrup M.C., Verweij P.E., Mouton J.W., Lagrou K., Meletiadis J. (2017). Multicentre validation of 4-well azole agar plates as a screening method for detection of clinically relevant azole-resistant *Aspergillus fumigatus*. J. Antimicrob. Ch..

[52] Zhang J., Snelders E., Zwaan B.J., Schoustra S.E., Meis J.F., van Dijk K., Hagen F., van der Beek M.T., Kampinga G.A., Zoll J. (2017). A Novel Environmental Azole Resistance Mutation in *Aspergillus fumigatus* and a Possible Role of Sexual Reproduction in Its Emergence. mBio.

[53] Watson A., Pung H., Hall B.H. (2017). Managing leaf diseases of carrots with traditional and alternative fungicides including baseline sensitivity studies. Crop. Protect..

[54] Bartlett D.W., Clough J.M., Godwin J.R., Hall A.A., Hamer M., Parr-Dobrzanski B. (2002). The strobilurin fungicides. Pest. Manag. Sci..

[55] Alshareef F., Robson G.D. (2014). Prevalence, persistence, and phenotypic variation of *Aspergillus fumigatus* in the outdoor environment in Manchester, UK, over a 2-year period. Med. Mycol..

[56] Shelton J.M.G., Fisher M.C., Singer A.C. (2020). Campaign-Based Citizen Science for Environmental Mycology: The “Science Solstice” and “Summer Soil-stice” Projects to Assess Drug Resistance in Air and Soilborne *Aspergillus fumigatus*. bioRxiv.

